# Highlight on Advances in Nontuberculous Mycobacterial Disease in North America

**DOI:** 10.1155/2014/919474

**Published:** 2014-09-11

**Authors:** Mehdi Mirsaeidi, Maham Farshidpour, Mary Beth Allen, Golnaz Ebrahimi, Joseph O. Falkinham

**Affiliations:** ^1^Section of Pulmonary, Critical Care, Sleep and Allergy, Department of Medicine M/C 719, University of Illinois at Chicago, 840 S. Wood Street, Chicago, IL 60612-7323, USA; ^2^Institute of Human Virology, University of Maryland School of Medicine, Baltimore, MD 21201, USA; ^3^Department of Health, University of Louisville, Louisville, KY 40202, USA; ^4^Department of Biological Science, University of Virginia Tech, Blacksburg, VA 24061, USA

## Abstract

Nontuberculous mycobacteria (NTM) are ubiquitous in the environment and exist as an important cause of pulmonary infections in humans. Pulmonary involvement is the most common disease manifestation of NTM and the incidence of NTM is growing in North America. Susceptibility to NTM infection is incompletely understood; therefore preventative tools are not well defined. Treatment of pulmonary nontuberculous mycobacterial (NTM) infection is difficult and entails multiple antibiotics and an extended treatment course. Also, there is a considerable variation in treatment management that should be considered before initiating treatment. We highlight the new findings in the epidemiology diagnosis and treatment of mycobacterial infections. We debate new advances regarding NTM infection in cystic fibrosis patients and solid organ transplant recipients. Finally, we introduce a new epidemiologic model for NTM disease based on virulence-exposure-host factors.

## 1. Introduction

Nontuberculous mycobacteria (NTM) are an important cause of morbidity in the United States. A few available prevalence studies show that NTM disease is increasing in the elderly population and suggesting NTM disease causes higher morbidity than TB in the US [1]. Patients with pulmonary NTM disease have significantly impaired health-related quality of life (HRQL) due to impaired lung function [2, 3]. The genus* Mycobacterium* includes over 150 species, many of which may cause disease [4]. Approximately 80% of pulmonary NTM (PNTM) infections in the United States are caused by members of the* Mycobacterium avium* complex (MAC) [5–7]. Molecular sequence data show that MAC includes 10 different subspecies such as* M. avium*,* M. hominissuis*,* M. silvaticum*, and* M. paratuberculosis, M. intracellulare*,* M*.* colombiense*,* M. bouchedurhonense*,* M. timonense*,* M. arosiense,* and* M. marseillense* [8].

Current published studies report that the prevalence of pulmonary NTM disease is rising throughout the United States, particularly among older adults [3, 9]. As the baby boomer cohort continues to age thus increasing the proportion of older Americans in the general population, it is expected that the incidence and prevalence of pulmonary NTM disease will likewise increase. Also, patients with NTM disease require frequent and intense healthcare resources such as hospitalizations and frequent office visits as well as complicated therapy and associated treatment challenges. These challenges are confounded when multiple comorbidities are also present, which are common in this population.

Many of the potential challenges with treating NTM infection in the US are offset by the improvement of medical knowledge over the last decade. This paper reviews important new developments in the prevalence, pathogenesis, diagnosis, and management of mainly pulmonary NTM disease in North America.

## 2. Methods

A literature search was conducted using search keywords “nontuberculous mycobacteria,” “MAC,” “*M. abscessus,”* “epidemiology,” “treatment,” “North America,” “mortality,” “cystic fibrosis,” “transplantation,” “prevention,” and “diagnosis” from studies that have been published between the years 2009 and 2014. PubMed, Cinahl, Scopus, Embase, and the Cochrane Library were searched. A total of 382 articles were reviewed from which 65 papers were selected that met our selection criteria. Titles of interest were further reviewed by all authors. Reference lists of relevant studies were hand-searched in order to identify other potentially relevant articles. Studies included in this review met the following criteria:

- study populations included patients with NTM;
- articles were full reports, case reports or reviews;
- articles were in English and published from the US based institutes;
- articles were published in peer-reviewed journals.

## 3. Epidemiology

Nontuberculous mycobacteria (NTM) are an important cause of morbidity and mortality, often in the form of progressive lung disease [5, 10, 11]. Few reports are accessible on NTM disease prevalence in the United States; however based on the recent data the incidence of pulmonary NTM has been reported to be rising in North America [3]. Winthrop et al. described the pulmonary NTM disease prevalence in the state of Oregon, USA [12]. The total age-adjusted prevalence of NTM was reported 8.6 per 100,000 population in the 2005-2006. However, 50 years of age and older had a higher rate of 20.4 per 100,000. The median age was 66 years and 59% were females [12]. In a combined report of four other regions in 2010, the mean annual prevalence was 5.5/100,000, ranging from 1.7/100,000 in Southern Colorado to 6.7/100,000 in Southern California [5]. Moreover, according to the national Medicare claims data by Adjemian et al., the annual prevalence of NTM in the population older than 65 years old significantly increased from 20 cases/100,000 persons in 1997 to 47 cases/100,000 persons in 2007, in which Caucasians account for 90% of cases followed by Asians/Pacific Islanders and Blacks [3, 13]. The prevalence of pulmonary nontuberculous mycobacterial disease differs by geographic region since specific environmental factors linked to water and soil exposure seem to increase the risk of PNTM infection. Adjemian et al. reported the 55 counties in 8 states with a particularly high risk of infection, including parts of California, New York, Florida, Hawaii, Louisiana, Oklahoma, Pennsylvania, and Wisconsin [14].

According to another study, NTM were found in 30% of patients with noncystic fibrosis bronchiectasis [15]. The frequency of NTM in the bronchiectasis population was 37%, 30% of which met the ATS criteria for NTM disease. MAC was the most common isolate (88%) found in this particular patient population [15].

In Ontario, Canada, the population cohort study showed that the NTM isolation prevalence raised from 9.1/100,000 in 1997 to 14.1/100,000 in 2003 [16]. Furthermore, Damaraju et al. found 10.8% patients with culture-proven pulmonary tuberculosis (PTB) in Ontario had NTM coisolated, including* Mycobacterium avium* complex (55%),* M. xenopi*, (18%), and* M*.* gordonae* (15%) [17].

## 4. Extrapulmonary NTM

Although the incidence of extrapulmonary NTM in the US remained largely unknown, it has been reported that up to 10% of NTM disease manifests as extrapulmonary [18]. The incidence of extrapulmonary NTM may be higher than our current estimation. NTM have potential to involve any human body organ and are commonly isolated from skin and soft tissue, lymphadenitis, septic arthritis, bone, and as disseminated infection [19–21]. A high index of clinical suspicion of disease and isolation of NTM from sterile site or any NTM growth from biopsy or compatible histopathology with mycobacterial disease are main keys to diagnose extrapulmonary NTM. A recently published study on 42 patients with confirmed NTM infection in upper extremity showed that there was a significant diagnosis delay due to its indolent presentation and lack of physician suspicion [22].  Table 2 shows nontuberculous mycobacteria strains associated with osteoarticular infections and skin diseases.

## 5. NTM in Elderly

According to a review conducted by Mirsaeidi et al., older people are at an increased risk for developing NTM infections and are most likely to use significant health care resources including long-term care services to manage NTM infections [23]. Given the aging of the US people and the incidence and severity of NTM disease in the elderly population, an increasing focus on research in the area of NTM including highly valid studies in the elderly should be considered. Another important factor when treating this population is therapy considerations given comorbidities and associated concomitant therapies. For this reason, drug-drug interaction is an important issue in elderly population. This is especially true regarding macrolides, rifamycins, and fluoroquinolones that are commonly used for NTM treatment [24, 25]. These treatment regimens usually cause interaction with the metabolisms of other drugs via interacting with cytochrome P-450 [25].

## 6. Mortality

United States population-based data demonstrate that the number of deaths from nontuberculous mycobacterial disease is growing. During the years 1999 through 2010, NTM disease was reported as an immediate cause of death in 2,990 people in the United States with a combined overall mean age-adjusted mortality rate of 0.1 per 100,000 person-years. Persons aged 55 years and older, women, those living in Hawaii and Louisiana, and those of non-Hispanic, white ethnicity had higher mortality rates. The majority of NTM deaths were reported in the hospital setting [34]. Additionally, there is a strong association between age and NTM mortality, which was found to be significantly higher in patients older than 65 years. In addition to the presence of comorbidities common in this population, advanced age itself was determined to be a strong predictor of mortality [34, 35].

## 7. Pathogenesis and Risk Factors

**Figure 1:**
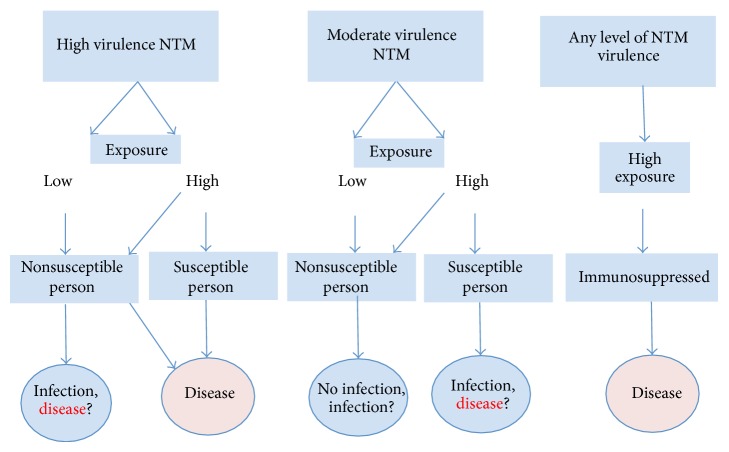
Illustrates our proposed virulence-exposure-host model for NTM disease. Virulence: High virulence NTM carries virulence antigens, although those antigens are largely unknown. Susceptible patient is defined as a person with chest wall abnormality, anatomical lung abnormalities, such as bronchiectasis, COPD, and asthma, and minor immune system abnormalities such as Mendelian susceptibility to mycobacterial disease. Infection: infection is defined as epithelial colonization by NTM without any evidence of tissue invasion including clinical and radiological evidence. Immunosuppressed patient is defined as a person with active malignancy except skin basal cell carcinoma on chemotherapy medication(s) and radiotherapy and HIV/AIDS, significant primary immunodeficiency, and corticosteroids therapy.

Everyone is virtually exposed to NTM, although most do not develop clinical signs of infection. The factors predisposing one to infection are not well described, but likely result from interaction between host defense mechanisms and the load of exposure [13].  Figure 1 illustrates our proposed epidemiologic model for NTM disease based on virulence-exposure-host factors. The infectious dose for NTM infection is largely unknown. It has been estimated that 10-10^2^ *M. bovis* organisms can cause pulmonary disease [36]. In mouse model for* M. ulcerans *infections an infectious dose of 10^3^-10^4^ colony-forming units are sufficient to induce swelling [37]. However, this data have never been extrapolated to other NTM species and also for humans.

Although for this reason NTM are considered opportunistic pathogens, they frequently cause infection in patients with no known underlying diseases. Even in seemingly normal hosts, some level of immunodeficiency or preexistent pulmonary disease probably exists [38]. Four categories of susceptible persons for NTM infection have been identified [23]. First, structural or preexisting pulmonary diseases such as cystic fibrosis, chronic obstructive pulmonary disease (COPD), and bronchiectasis have been strongly associated with the risk of developing several infectious lung diseases including NTM. Second, patients with autoimmune disorders who are being treated with antitumor necrosis factor-*α* (TNF-*α*) drugs are at risk for developing NTM as well as many other opportunistic infections. Third, HIV infected persons with AIDS are also at an increased risk for developing NTM along with many other opportunistic infections. In fact, a CD4+ T cell count of less than 50 cells/*μ*L is associated with increased risk of disseminated NTM disease. Fourth, patients with genetic syndromes involving mutations in the interleukin-12 or interferon *γ* pathways are also at risk for developing opportunistic infections including NTM. Mutations in these pathways are associated with both autoimmune disorders as well as immune suppression [39–42]. Additionally, nonsmoker elderly females with a slender body and some with characteristic features such as scoliosis, pectus defects, or mitral valve prolapsed are more prone to pulmonary NTM compared to the normal population [15, 43, 44]. The last group forms the majority of patients that are seen in our practice in Chicago.

There are limited data on the genetic susceptibility to NTM infection. The familial clustering of pulmonary NTM infections has only been rarely reported [45]. There is some evidence for association between NTM disease and natural resistance-associated macrophage protein 1 gene (NRAMP1) [46]. NRAMP1 regulates intramacrophage iron concentrations to limit the availability of iron for intracellular bacteria [47], as demonstrated in* Mycobacterium bovis* residing within the phagolysosome [47, 48].

## 8. NTM and Organ Transplantation

Solid organ transplant recipients could also have increased risk of NTM disease for several reasons. Posttransplantation immunosuppressive therapy may increase the likelihood of clinical disease from environmental exposures [49]. Also, underlying lung disease in lung transplant patients could place patients at a higher risk for NTM infection during the pretransplant period. Possible risk factors for reinfection or new disease with NTM after lung transplantation are immunosuppression and the development of structural lung disease over time secondary to bronchiolitis obliterans syndrome [50, 51]. Longworth et al. reported 34 cases of solid organ patients with NTM, which were predominantly males with a median age of 55 years with disease incidence following a median of 8 months after transplantation.* Mycobacterium abscessus* and* Mycobacterium avium complex* were the most common pathogens, and the lung (including pleura) was the most common site of disease. In this adjusted case-control analysis, lung transplant recipients had the highest risk of NTM disease [52]. According to Knoll et al., NTM were isolated from 53 of 237 patients (22.4%) following lung transplantation over a median of 25.2 months follow-up. The incidence rate of NTM isolation was 9.0/100 person-years, and the incidence rate of NTM disease was 1.1/100 person-years. The most common NTM isolated was MAC (69.8%), followed by* M. abscessus* (9.4%) and* M. gordonae* (7.5%) [51]. Huang et al. found out NTM infection notably increased the risk of death after lung transplantation (HR = 2.61, *P* = 0.001) following an assessment of 201 primary lung transplant recipients transplanted between January 2000 and August 2006. The increased risk was observed for both NTM colonization and NTM disease [53].

## 9. NTM and Cystic Fibrosis

Cystic fibrosis (CF) possesses a strong association with NTM for a number of reasons. First, the underlying lung problems characteristic of CF put patients with this disease at a unique risk for developing NTM following exposure. Also, the increasing lifespan of CF patients secondary to improvements in management places CF patients at a longer lifetime risk for developing NTM infection as compared to the general population [42]. NTM could be present intermittently in low quantities in the airways of CF patients. NTM have been isolated from up to 32% of CF patients [54]. Although the effect of chronic and recurrent NTM infection in the CF course is not clear, it is quite possible that progressive respiratory decline because NTM disease may also affect CF disease outcomes. Identifying NTM in CF patient is rather difficult for clinicians given the common symptoms exhibited by CF patients without NTM [55]. Although NTM are usually not believed to be a transmissible disease, current evidence by Aitken et al. documented an outbreak of* M. abscessus* subspecies* massiliense* with similar genome sequencing in five CF patients at the University of Washington. This report has brought to light the possibility that* M*. abscessus can indeed be transmitted among CF populations [56]. In 2010, Esther et al. reported microbiological data from 1216 CF patients demonstrating that chronic* M. abscessus* infection was associated with clinical deterioration as measured by an increased rate of decline in FEV1 [57].

## 10. NTM and TNF-*α*

The therapeutic use of TNF-*α* receptor antagonist drugs, particularly in rheumatoid arthritis and other connective tissue disorders patients, is a risk factor for NTM infection. In a review of 8418 anti-TNF-*α* users, Winthrop et al. reported that 18 cases developed NTM and 16 individuals were diagnosed with tuberculosis after drug initiation. The rates (per 100,000 person-years) for NTM, respectively, for etanercept were 35 (95% CI: 1 to 69), infliximab were 116 (95% CI: 30 to 203), and adalimumab were 122 (95% CI: 3 to 241) [40]. Most cases of NTM infections were pulmonary (67%), but there were considerable (22%) extrapulmonary sites of involvement as well.* M. avium* was accountable for half of the cases and in a review of 8,000 users of anti-TNF-*α* medications the rate of NTM was 74/100,000 person years [40, 58]. The same group reported that* M. avium* (49%) following rapidly growing mycobacteria (19%) were the most common etiologic microorganism in anti-TNF-*α* receivers [40].

## 11. Diagnosis

The diagnosis of NTM infection can be quite challenging. First, culturing NTM can be tricky because the bacteria are ubiquitous in the environment and may contaminate clinical samples from nonsterile sites. Contamination may occur before, during, and even after sampling. For example, collected sputum samples may be contaminated if rinsed in the mouth with tap water before expectoration [59]. Fibrotic bronchoscope suction channel contamination with* Mycobacterium chelonae* has also been reported as a cause of pseudoepidemic [60]. In order to distinguish between contamination and infection, a diagnosis of NTM pulmonary disease should be established in a combination of clinical, radiological, bacteriological, and histological criteria [39, 59, 61]. A clinical and radiological diagnostic criteria overview is outside of scope of this review and could be found elsewhere [23].

## 12. Methods Used for the Detection of NTM

### 12.1. Staining and Culture

Smear staining is routinely performed in a two-step procedure. First, samples are screened by fluorochrome (auramine) staining due to the high sensitivity and positives are confirmed by classical Ziehl-Neelsen staining [62]. Once preparing specimens for isolation, decontamination by N-acetyl-L-cysteine-sodium hydroxide (NALC/NaOH) is needed to prevent the growth of other bacteria; however, samples from patients with cystic fibrosis should be treated with an additional decontamination step with oxalic acid to diminish the Gram-negative overgrowth and increase the frequency of detection of NTM by culture [63]. In general, liquid media are more sensitive rather than solid media such as Lowenstein-Jensen [64]. The highest frequency of recovery of NTM is expected to be obtained if both solid and liquid media are applied and incubated at both 37 and 30°C if M. marinum is suspected [62]. Most NTM strains grow within 2 to 3 weeks with the exception of rapidly growing mycobacteria types like* M. abscessus, M. fortuitum, Mycobacterium chelonae,* and* M. massiliense,* which may grow within 7 days [65].

### 12.2. Molecular Methods

The methods for the identification of mycobacteria in clinical laboratories have improved considerably over the last 2 decades. Also, species identification offers an opportunity to further expand the clinical and epidemiologic database regarding NTM which may ultimately produce treatment trials and accurate outcome studies [66]. Current rapid techniques for the identification of NTM consist of probes, high-performance liquid chromatography (HPLC), and other molecular techniques [67]. HPLC recognizes mycobacteria according to variations in mycolic acids, the long-chain fatty acids resided in the cell wall of mycobacteria [68]. Molecular DNA probes have now been applied for identifying MAC,* M. gordonae*, and* M. kansasii*; however, this process is costly and probes are not provided for all species of mycobacteria [69]. Polymerase chain reaction (PCR) restriction fragment length polymorphism analysis is another molecular technique for identifying mycobacteria on account of differences in restriction fragments of the 65 kD heat-shock protein. Sequence analysis of the rpoB gene and 16S ribosomal RNA has been expanded recently as another method for speciation of NTM [70, 71]. A recent study proposed serodiagnosis of pulmonary NTM infection as a possible diagnostic method in order to identify antibodies specific to lipid antigen in NTM [72].

Reverse hybridization is a commonly used method in clinical laboratories to identify those NTM species uncovered by the Accuprobe assay [73]. Most species can be identified by using Genotype and Inno-Lipa diagnostic kits which are mainly used in Europe [74].

## 13. Drug Susceptibility Test

Most NTM infections are managed with antimicrobial agents. Consequently, the role of drug susceptibility testing (DST) on NTM isolates is critical in the determination of drug therapy regimens for NTM disease [75]. The current ATS/IDSA guidelines recommend drug susceptibility tests for MAC (macrolides),* M. kansasii* (rifampin), and rapid growing mycobacteria [59]. There are not enough data available regarding the role of DST in other species of NTM [76].

Most NTM strains are resistant to conventional antituberculous agents, leaving fewer options for treatment than many other diseases. Also, clarithromycin is along the most preferred agent in many cases if the isolate is susceptible, which further emphasizes the need for DST [59]. Recently, Babady et al. [77] discovered the clarithromycin susceptibility testing of MAC by the SLOMYCO panel and the JustOne strip methods are simple to set up and easy to interpret. BACTEC 460 system is a well-established assay for clarithromycin susceptibility testing of MAC isolates. The concordance between the SLOMYCO panel or the JustOne strip and the BACTEC 460 method was 90%, with the kappa score indicating sound agreement between the methods.

The JustOne strip and the SLOMYCO panel are both broth microdilution methods, and they exhibit ≥90% correlation with both the radiometric method and a broth microdilution reference method. Additionally, the SLOMYCO panel and the JustOne strip have the advantage of being commercially accessible and simple to set up and read and the susceptibility results are frequently available within 7 days. This is much quicker than the BACTEC 460 method, which also avoids the use of costly instrumentation and allows therapy to be initiated sooner [77, 78].

## 14. Treatment

The management of NTM infection is mainly by drug therapy. However, drug used to treat NTM disease is often expensive; the course is lengthy, and treatment is often correlated with drug-related toxicities [76, 79]. The treatment regimens vary by species with the most important distinction being that between slow* versus* rapid growing NTM [6] (Table 1). For most slow growing strains, the optional regimen includes rifampicin (Rifapentine or rifabutin) and ethambutol and a macrolide is administrated for 18–24 months; amikacin or streptomycin should be added in the initial 3–6 months in cases of severe disease. For the rapid growing strains, regimens are based on* in vitro* DST results. For* Mycobacterium abscessus*, these regimens usually consist of a macrolide, amikacin and either cefoxitin, imipenem, or tigecycline [75, 80]. Jarand et al. reported the management results for* M. abscessus* pulmonary disease patients who received antibiotic treatment that was individualized according to patient tolerance and drug susceptibility outcomes. Sixteen different antibiotics were administrated with forty-two different combinations for an average of 4.6 drugs per patient over the course of a median of 6 months. Forty-nine patients converted sputum cultures to negative, but 16 (23%) experienced relapse later [80]. Additionally, Safdar showed that aerosolized amikacin with a range of 7,600 to 95,400 mg was effective in the treatment of eight PNTM patients who previously failed combination oral drug therapy [81]. Patients with anti-IFN-*γ* autoantibodies (a rare underlying disease for NTM) have impaired IFN-*γ* signaling which may lead to severe disseminated infections with intracellular pathogens including primarily NTM [82, 83]. Rituximab has no role in the treatment of NTM disease except this rare condition. Browne et al. used rituximab (anti CD-20) in 4 patients with disseminated infection with* Mycobacterium abscessus*,* M. avium*, and* M. intracellulare* due to high-titer anti-IFN-*γ* autoantibodies. All subjects had received ≥3 antimycobacterial agents before rituximab treatment. Rituximab was given at 375 mg/m^2^ weekly for ≥4 doses and then at wider intervals. All patients received between 8 and 12 doses over the first year with subsequent additional doses determined by the recurrence of infection. Within 2–6 months after initiation of the rituximab treatment, all patients had marked clinical, radiologic, and laboratory improvement [84]. Moreover, one more case of anti-IFN-*γ* autoantibody syndrome with disseminated infection by* M. abscessus* was successfully treated with rituximab at a dose of 375 mg/m^2^ by Czaja et al. [85].

Cure rates of pulmonary NTM disease is different by species, ranging from 30–50% in* M. abscessus* disease to 50–70% in* Mycobacterium avium* complex and 80–90% in* Mycobacterium malmoense* and* Mycobacterium kansasii* disease [80, 86]. According an* in vitro* study by Van Ingen et al., clofazimine and amikacin illustrated significant synergistic activity against a variety of NTM, including both slow and rapid growing strains. This* in vitro* study consisted mostly of MAC,* M*.* abscessus*, and* M. simiae*, which are all well-known causative agents of human disease with challenging drug treatment options and inferior clinical outcomes [87]. Regarding MAC management, Wallace et al. recently demonstrated that among 180 cases with nodular/bronchiectatic (NB) MAC lung disease, treatment with macrolide/azalide-containing regimens such as clarithromycin or azithromycin may lead to 84% successful sputum conversion without true microbiologic relapse. Interestingly, no patient developed macrolide resistance during treatment and intermittent therapy was effective and considerably better tolerated than daily therapy [88]. On the other hand, long-term monotherapy with azithromycin in 191 persons with CF appeared to be associated with a lower frequency of incident NTM infections. However, since macrolide monotherapy could lead to macrolide resistance, routine screening for NTM should be considered for persons with CF [89].

A new report has documented that tigecycline as part of a multidrug regimen resulted in improvement in >60% of 52 patients with* M. abscessus* and* M. chelonae* infections, including those with underlying cystic fibrosis despite having failed prior antibiotic therapy. However, adverse events with tigecycline were reported in >90% of cases, the most common being nausea and vomiting [90].

Indications for surgery have not been uniformly accepted although would be considered in the events of medication intolerance, drug resistance, and/or localized cavitation. Other indications include recurrent or massive hemoptysis and the presence of a destroyed lung. In experienced hands and careful patient selection, the safety of lung resection for NTM lung disease, particularly thoracoscopic right middle lobe lobectomy and lingulectomy, seems good [79]. Data have shown that surgery provides improved microbiologic response for refractory organisms such as* M. abscessus* as compared to medication regimens alone [79, 80, 91].

## 15. Cost of Treatment

The treatment of pulmonary nontuberculous mycobacterial (NTM) infection is difficult and entails multiple antibiotics and an extended treatment course. However, limited data are available regarding the cost of NTM treatment in the United States. Leber and Marras determined the monthly mean cost of treating 91 patients with pulmonary NTM infections in a tertiary care facility in Toronto, Ontario, Canada was equal to 292 US dollars (USD). The median total duration and cost per treated patient were 14 months (interquartile range (IQR) 9–23 months) and 4,484 USD, correspondingly. The most costly oral regiment includes rifampin in addition to fluroquinolone and macrolide [92]. Collier et al. reported that the direct cost of inpatient treatment for NTM was $21,041 per each episode of admission [93].

## 16. Conclusions

The incidence of NTM infection is growing in North America. In addition to population distribution factors resulting in more elderly Americans coupled with the higher incidence of disease occurring in this population placing more people at risk for disease following exposure. Also, more immunosuppressed patients of all ages are likewise susceptible to disease often due to medical advances in treating autoimmune disorder, HIV/AIDS, and the availability of solid organ transplantation as an option for treating a myriad of diseases. In addition to disease susceptibility, diagnostics of NTM have demonstrated remarkable improvement allowing cases to be better identified. These improvements include liquid culture techniques and advance molecular methods. Despite these significant advances over the last few years, susceptibility to disease is incompletely recognized. This is an undermined effort to identify a complete understanding of at risk populations and determine preventative tools. Additionally, given the difficulty of eradicating NTM and its considerable reoccurrence, identifying appropriate candidates for treatment and the timing of initiation of therapy are likewise challenging. There is a considerable variation in treatment management that should be deliberated before initiation. While the US populations are aging and NTM diseases are rising in elderly population, we would hope to see an increasing focus on research in NTM infection and multicenter trials. It is critical that this condition is recognized as an important public health issue with potentially significant consequences for affected patients. Finally, the applicability of the virulence-exposure-host model in NTM disease should be investigated.

## Figures and Tables

**Table 1:** The most common NTM species isolated from patients in North America.

Slow-growing mycobacteria (SGM)	Rapid-growing mycobacteria (RGM)
*M. avium complex *	*M. abscessus *
*M. kansasii *	*M. chelonae *
*M. xenopi *	*M. fortuitum *
*M. simiae *	*M. marinum *
*M. malmoense *	
*M. szulgai *	

**Table 2:** Some of extrapulmonary NTM diseases reported from skin, soft tissue, bone, and joints.

Clinical presentation	Mycobacterium species	Comorbidities	References
Arthritis	*M. Chelonae * *MAC * *M. fortuitum* *M. marinum *	Rheumatoid arthritis	[26]

Tenosynovitis	*MAC* *M. chelonae *	Bone fracture, penetrating injury	[27]

Osteomyelitis	*M. szulgai* *M. abscessus* *M. fortuitum* *M. chelonae*	Inherited STAT1 deficiency, hepatitis C, former intravenous drug user, none	[28–31]

Skin and soft tissue	*M. chelonae* *M. marinum* *M. avium *	Tattoos None None	[32, 33]

## References

[1] Cassidy P. M., Hedberg K., Saulson A., McNelly E., Winthrop K. L. (2009). Nontuberculous mycobacterial disease prevalence and risk factors: a changing epidemiology. *Clinical Infectious Diseases*.

[2] Mehta M., Marras T. K. (2011). Impaired health-related quality of life in pulmonary nontuberculous mycobacterial disease. *Respiratory Medicine*.

[3] Adjemian J., Olivier K. N., Seitz A. E., Holland S. M., Prevots D. R. (2012). Prevalence of nontuberculous mycobacterial lung disease in U.S. medicare beneficiaries. *The American Journal of Respiratory and Critical Care Medicine*.

[4] Tortoli E. (2003). Impact of genotypic studies on mycobacterial taxonomy: the new mycobacteria of the 1990s. *Clinical Microbiology Reviews*.

[5] Prevots D. R., Shaw P. A., Strickland D., Jackson L. A., Raebel M. A., Blosky M. A., De Oca R. M., Shea Y. R., Seitz A. E., Holland S. M., Olivier K. N. (2010). Nontuberculous mycobacterial lung disease prevalence at four integrated health care delivery systems. *American Journal of Respiratory and Critical Care Medicine*.

[6] Kim C. J., Kim N. H., Song K. H., Choe P. G., Kim E. S., Park S. W., Kim H. B., Kim N. J., Kim E. C., Park W. B., Oh M. D. (2013). Differentiating rapid- and slow-growing mycobacteria by difference in time to growth detection in liquid media. *Diagnostic Microbiology and Infectious Disease*.

[7] Machado D., Ramos J., Couto I., Cadir N., Narciso I., Coelho E., Viegas S., Viveiros M. (2014). Assessment of the BD MGIT TBc identification test for the detection of *Mycobacterium tuberculosis* complex in a network of mycobacteriology laboratories. *BioMed Research International*.

[8] Cayrou C., Turenne C., Behr M. A., Drancourt M. (2010). Genotyping of Mycobacterium avium complex organisms using multispacer sequence typing. *Microbiology*.

[9] Billinger M. E., Olivier K. N., Viboud C., Montes De Oca R., Steiner C., Holland S. M., Prevots D. R. (2009). Nontuberculous mycobacteria-associated lung disease in hospitalized persons, United States, 1998–2005. *Emerging Infectious Diseases*.

[10] van Ingen J., Ferro B. E., Hoefsloot W., Boeree M. J., van Soolingen D. (2013). Drug treatment of pulmonary nontuberculous mycobacterial disease in HIV-negative patients: the evidence. *Expert Review of Anti-Infective Therapy*.

[11] Griffith D. E. (2010). Nontuberculous mycobacterial lung disease. *Current Opinion in Infectious Diseases*.

[12] Winthrop K. L., McNelley E., Kendall B., Marshall-Olson A., Morris C., Cassidy M., Saulson A., Hedberg K. (2010). Pulmonary nontuberculous mycobacterial disease prevalence and clinical features: an emerging public health disease. *The American Journal of Respiratory and Critical Care Medicine*.

[13] Margaret M., Johnson J. A. O. (2014). Nontuberculous mycobacterial pulmonary infections. *Journal of Thoracic Disease*.

[14] Adjemian J., Olivier K. N., Seitz A. E., Falkinham J. O., Holland S. M., Prevots D. R. (2012). Spatial clusters of nontuberculous mycobacterial lung disease in the United States. *American Journal of Respiratory and Critical Care Medicine*.

[15] Mirsaeidi M., Hadid W., Ericsoussi B., Rodgers D., Sadikot R. T. (2013). Non-tuberculous mycobacterial disease is common in patients with non-cystic fibrosis bronchiectasis. *International Journal of Infectious Diseases*.

[16] Marras T. K., Mehta M., Chedore P., May K., Houqani M. A., Jamieson F. (2010). Nontuberculous mycobacterial lung infections in Ontario, Canada: Clinical and microbiological characteristics. *Lung*.

[17] Damaraju D., Jamieson F., Chedore P., Marras T. K. (2013). Isolation of non-tuberculous mycobacteria among patients with pulmonary tuberculosis in Ontario, Canada. *International Journal of Tuberculosis and Lung Disease*.

[18] Kasperbauer S., Huitt G. (2013). Management of extrapulmonary nontuberculous mycobacterial infections. *Seminars in Respiratory and Critical Care Medicine*.

[19] Parker N. P., Scott A. R., Finkelstein M., Tibesar R. J., Lander T. A., Rimell F. L., Sidman J. D. (2012). Predicting surgical outcomes in pediatric cervicofacial nontuberculous mycobacterial lymphadenitis. *Annals of Otology, Rhinology & Laryngology*.

[20] Laquer V., Ta T., Nguyen T., Tan B. (2013). Mycobacterium poriferae infection in a psoriasis patient on anti-TNF-*α* therapy. *Dermatology Online Journal*.

[21] Lembo G., Goldstein E. J. C., Troum O., Mandelbaum B. (2012). Successful treatment of mycobacterium terrae complex infection of the knee. *Journal of Clinical Rheumatology*.

[22] Al-Knawya W. B., Garnerb H., Mirsaeidic M., Cawleyd J., Murraye P., Brumblea L., Alvareza S. (2014). A descriptive analysis of nontuberculous mycobacterial infections (NTM) of the upper extremity. *International Journal of Mycobacteriology*.

[23] Mirsaeidi M., Farshidpour M., Ebrahimi G., Aliberti S., Falkinham J. O. (2014). Management of nontuberculous mycobacterial infection in the elderly. *European Journal of Internal Medicine*.

[24] Mergenhagen K. A., Olbrych P. M., Mattappallil A., Krajewski M. P., Ott M. C. (2013). Effect of azithromycin on anticoagulation-related outcomes in geriatric patients receiving warfarin. *Clinical Therapeutics*.

[25] Baciewicz A. M., Chrisman C. R., Finch C. K., Self T. H. (2013). Update on rifampin, rifabutin, and rifapentine drug interactions. *Current Medical Research and Opinion*.

[26] Brode S. K., Jamieson F. B., Ng R., Campitelli M. A., Kwong J. C., Paterson J. M., Li P., Marchand-Austin A., Bombardier C., Marras T. K. (2014). Risk of mycobacterial infections associated with rheumatoid arthritis in Ontario, Canada. *Chest*.

[27] Piersimoni C., Scarparo C. (2009). Extrapulmonary infections associated with nontuberculous mycobacteria in immunocompetent persons. *Emerging Infectious Diseases*.

[28] Shamriz O., Engelhard D., Rajs A. P., Kaidar-Shwartz H., Casanova J.-L., Averbuch D. (2013). Mycobacterium szulgai chronic multifocal osteomyelitis in an adolescent with inherited STAT1 deficiency. *Pediatric Infectious Disease Journal*.

[29] Garcia D. C., Sandoval-Sus J., Razzaq K., Young L. (2013). Vertebral osteomyelitis caused by Mycobacterium abscessus. *BMJ Case Reports*.

[30] Longardner K., Allen A., Ramgopal M. (2013). Spinal osteomyelitis due to *Mycobacterium fortuitum* in a former intravenous drug user. *BMJ Case Reports*.

[31] Talanow R., Vieweg H., Andresen R. (2013). Atypical osteomyelitis caused by mycobacterium chelonae—a multimodal imaging approach. *Case Reports in Infectious Diseases*.

[32] Kennedy B. S., Bedard B., Younge M., Tuttle D., Ammerman E., Ricci J., Doniger A. S., Escuyer V. E., Mitchell K., Noble-Wang J. A., O'Connell H. A., Lanier W. A., Katz L. M., Betts R. F., Mercurio M. G., Scott G. A., Lewis M. A., Goldgeier M. H. (2012). Outbreak of *Mycobacterium chelonae* infection associated with tattoo ink. *The New England Journal of Medicine*.

[33] Pham-Huy A., Robinson J. L., Tapiéro B., Bernard C., Daniel S., Dobson S., Déry P., Le Saux N., Embree J., Valiquette L., Quach C. (2010). Current trends in nontuberculous mycobacteria infections in Canadian children: a Pediatric Investigators Collaborative Network on Infections in Canada (PICNIC) study. *Paediatrics and Child Health*.

[34] Mirsaeidi M., Machado R. F., Garcia J. G. N., Schraufnagel D. E. (2014). Nontuberculous mycobacterial disease mortality in the United States, 1999-2010: a population-based comparative study. *PLoS ONE*.

[35] Andréjak C., Thomsen V. Ø., Johansen I. S., Riis A., Benfield T. L., Duhaut P., Sørensen H. T., Lescure F.-X., Thomsen R. W. (2010). Nontuberculous pulmonary mycobacteriosis in Denmark: incidence and prognostic factors. *The American Journal of Respiratory and Critical Care Medicine*.

[36] O'Reilly L. M., Daborn C. J. (1995). The epidemiology of Mycobacterium bovis infections in animals and man: a review. *Tubercle and Lung Disease*.

[37] Dega H., Robert J., Bonnafous P., Jarlier V., Grosset J. (2000). Activities of several antimicrobials against Mycobacterium ulcerans infection in mice. *Antimicrobial Agents and Chemotherapy*.

[38] Mirsaeidi M. (2012). Personalized medicine approach in mycobacterial disease. *International Journal of Mycobacteriology*.

[39] Keating M. R., Daly J. S. (2013). Nontuberculous mycobacterial infections in solid organ transplantation. *American Journal of Transplantation*.

[40] Winthrop K. L., Chang E., Yamashita S., Iademarco M. F., LoBue P. A. (2009). Nontuberculous mycobacteria infections and anti-tumor necrosis factor-*α* therapy. *Emerging Infectious Diseases*.

[41] Chan E. D., Iseman M. D. (2013). Underlying host risk factors for nontuberculous mycobacterial lung disease. *Seminars in Respiratory and Critical Care Medicine*.

[42] Leung J. M., Olivier K. N. (2013). Nontuberculous mycobacteria: The changing epidemiology and treatment challenges in cystic fibrosis. *Current Opinion in Pulmonary Medicine*.

[43] Kartalija M., Ovrutsky A. R., Bryan C. L., Pott G. B., Fantuzzi G., Thomas J., Strand M. J., Bai X., Ramamoorthy P., Rothman M. S., Nagabhushanam V., McDermott M., Levin A. R., Frazer-Abel A., Giclas P. C., Korner J., Iseman M. D., Shapiro L., Chan E. D. (2013). Patients with nontuberculous mycobacterial lung disease exhibit unique body and immune phenotypes. *The American Journal of Respiratory and Critical Care Medicine*.

[44] Chan E. D., Iseman M. D. (2010). Older women appear to be more susceptible to nontuberculous mycobacterial lung disease. *Gender Medicine*.

[45] Colombo R. E., Hill S. C., Claypool R. J., Holland S. M., Olivier K. N. (2010). Familial clustering of pulmonary nontuberculous mycobacterial disease. *Chest*.

[46] Sapkota B. R., Hijikata M., Matsushita I., Tanaka G., Ieki R., Kobayashi N., Toyota E., Nagai H., Kurashima A., Tokunaga K., Keicho N. (2012). Association of SLC11A1 (NRAMP1) polymorphisms with pulmonary Mycobacterium avium complex infection. *Human Immunology*.

[47] Weiss G., Fritsche G., Nairz M., Libby S. J., Fang F. C. (2012). Slc11a1 (Nramp1) impairs growth of *Salmonella enterica* serovar typhimurium in macrophages via stimulation of lipocalin-2 expression. *Journal of Leukocyte Biology*.

[48] Zwilling B. S., Kuhn D. E., Wikoff L., Brown D., Lafuse W. (1999). Role of iron in Nramp1-mediated inhibition of mycobacterial growth. *Infection and Immunity*.

[49] Dorman S., Subramanian A. (2009). Nontuberculous mycobacteria in solid organ transplant recipients. *The American Journal of Transplantation*.

[50] Piersimoni C. (2012). Nontuberculous mycobacteria infection in solid organ transplant recipients. *European Journal of Clinical Microbiology and Infectious Diseases*.

[51] Knoll B. M., Kappagoda S., Gill R. R., Goldberg H. J., Boyle K., Baden L. R., Fuhlbrigge A. L., Marty F. M. (2012). Non-tuberculous mycobacterial infection among lung transplant recipients: a 15-year cohort study. *Transplant Infectious Disease*.

[52] Longworth S. A., Vinnard C., Lee I., Sims K. D., Barton T. D., Blumberg E. A. (2014). Risk factors for nontuberculous mycobacterial infections in solid organ transplant recipients: a case-control study. *Transplant Infectious Disease*.

[53] Huang H. C., Weigt S. S., Derhovanessian A., Palchevskiy V., Ardehali A., Saggar R., Saggar R., Kubak B., Gregson A., Ross D. J., Lynch J. P., Elashoff R., Belperio J. A. (2011). Non-tuberculous mycobacterium infection after lung transplantation is associated with increased mortality. *Journal of Heart and Lung Transplantation*.

[54] Rodman D. M., Polis J. M., Heltshe S. L., Sontag M. K., Chacon C., Rodman R. V., Brayshaw S. J., Huitt G. A., Iseman M. D., Saavedra M. T., Taussig L. M., Wagener J. S., Accurso F. J., Nick J. A. (2005). Late diagnosis defines a unique population of long-term survivors of cystic fibrosis. *American Journal of Respiratory and Critical Care Medicine*.

[55] Leung J. M., Olivier K. N. (2013). Nontuberculous mycobacteria in patients with cystic fibrosis. *Seminars in Respiratory and Critical Care Medicine*.

[56] Aitken M. L., Limaye A., Pottinger P., Whimbey E., Goss C. H., Tonelli M. R., Cangelosi G. A., Ashworth Dirac M., Olivier K. N., Brown-Elliott B. A., McNulty S., Wallace R. J. (2012). Respiratory outbreak of Mycobacterium abscessus subspecies massiliense in a lung transplant and cystic fibrosis center. *The American Journal of Respiratory and Critical Care Medicine*.

[57] Esther C. R., Esserman D. A., Gilligan P., Kerr A., Noone P. G. (2010). Chronic *Mycobacterium abscessus* infection and lung function decline in cystic fibrosis. *Journal of Cystic Fibrosis*.

[58] Winthrop K. L., Baxter R., Liu L., Varley C. D., Curtis J. R., Baddley J. W., McFarland B., Austin D., Radcliffe L., Suhler E. B., Choi D., Rosenbaum J. T., Herrinton L. J. (2013). Mycobacterial diseases and antitumour necrosis factor therapy in USA. *Annals of the Rheumatic Diseases*.

[59] Griffith D. E., Aksamit T., Brown-Elliott B. A., Catanzaro A., Daley C., Gordin F., Holland S. M., Horsburgh R., Huitt G., Iademarco M. F., Iseman M., Olivier K., Ruoss S., Von Reyn C. F., Wallace R. J., Winthrop K. (2007). An official ATS/IDSA statement: diagnosis, treatment, and prevention of nontuberculous mycobacterial diseases. *The American Journal of Respiratory and Critical Care Medicine*.

[60] Wang H. C., Liaw Y. S., Yang P. C., Kuo S. H., Luh K. T. (1995). A pseudoepidemic of Mycobacterium chelonae infection caused by contamination of a fibreoptic bronchoscope suction channel. *European Respiratory Journal*.

[61] Arend S. M., Van Soolingen D., Ottenhoff T. H. (2009). Diagnosis and treatment of lung infection with nontuberculous mycobacteria. *Current Opinion in Pulmonary Medicine*.

[62] van Ingen J. (2013). Diagnosis of nontuberculous mycobacterial infections. *Seminars in Respiratory and Critical Care Medicine*.

[63] de Bel A., de Geyter D., de Schutter I., Mouton C., Wellemans I., Hanssens L., Schelstraete P., Malfroot A., Pierard D. (2013). Sampling and decontamination method for culture of Nontuberculous Mycobacteria in respiratory samples of cystic fibrosis patients. *Journal of Clinical Microbiology*.

[64] Chihota V. N., Grant A. D., Fielding K., Ndibongo B., Van Zyl A., Muirhead D., Churchyard G. J. (2010). Liquid vs. solid culture for tuberculosis: performance and cost in a resource-constrained setting. *International Journal of Tuberculosis and Lung Disease*.

[65] Saleeb P., Olivier K. N. (2010). Pulmonary nontuberculous mycobacterial disease: new insights into risk factors for susceptibility, epidemiology, and approaches to management in immunocompetent and immunocompromised patients. *Current Infectious Disease Reports*.

[66] Park J. S., Choi J.-I., Lim J.-H., Ahn J.-J., Jegal Y., Seo K. W., Ra S. W., Jeon J. B., Lee S. H., Kim S.-R., Jeong J. (2013). The combination of real-time PCR and HPLC for the identification of non-tuberculous mycobacteria. *Annals of Laboratory Medicine*.

[67] Jagielski T., Van Ingen J., Rastogi N., Dziadek J., Mazur P. K., Bielecki J. (2014). Current methods in the molecular typing of *Mycobacterium tuberculosis* and other Mycobacteria. *BioMed Research International*.

[68] Jeong J., Kim S.-R., Lee S. H., Lim J.-H., Choi J. I., Park J. S., Chang C. L., Choi J. Y., Richman D. D., Smith D. M. (2011). The use of high performance liquid chromatography to speciate and characterize the epidemiology of mycobacteria. *Laboratory Medicine*.

[69] Rogers J. T., Procop G. W., Steelman C. K., Abramowsky C. R., Tuohy M. T., Shehata B. M. (2012). Clinical utility of DNA amplification and sequencing to identify a strain of Mycobacterium avium in paraffin-embedded, formalin-fixed biopsies from an immunosuppressed child. *Pediatric and Developmental Pathology*.

[70] Varma-Basil M., Garima K., Pathak R., Dwivedi S. K. D., Narang A., Bhatnagar A., Bose M. (2013). Development of a novel pcr restriction analysis of the hsp65 gene as a rapid method to screen for the *Mycobacterium tuberculosis* complex and nontuberculous mycobacteria in high-burden countries. *Journal of Clinical Microbiology*.

[71] Jang M.-A., Koh W.-J., Huh H. J., Kim S.-Y., Jeon K., Ki C.-S., Lee N. Y. (2014). Distribution of nontuberculous mycobacteria by multigene sequence-based typing and clinical significance of isolated strains. *Journal of Clinical Microbiology*.

[72] Shin A.-R., Lee K.-S., Lee K. I., Shim T. S., Koh W.-J., Jeon H. S., Son Y.-J., Shin S.-J., Kim H.-J. (2013). Serodiagnostic potential of Mycobacterium avium MAV2054 and MAV5183 proteins. *Clinical and Vaccine Immunology*.

[73] Somoskovi A., Mester J., Hale Y. M., Parsons L. M., Salfinger M. (2002). Laboratory diagnosis of nontuberculous mycobacteria. *Clinics in Chest Medicine*.

[74] Tortoli E., Mariottini A., Mazzarelli G. (2003). Evaluation of INNO-LiPA MYCOBACTERIA v2: improved reverse hybridization multiple DNA probe assay for mycobacterial identification. *Journal of Clinical Microbiology*.

[75] van Ingen J., Boeree M. J., van Soolingen D., Mouton J. W. (2012). Resistance mechanisms and drug susceptibility testing of nontuberculous mycobacteria. *Drug Resistance Updates*.

[76] Brown-Elliott B. A., Nash K. A., Wallace R. J. (2012). Antimicrobial susceptibility testing, drug resistance mechanisms, and therapy of infections with nontuberculous mycobacteria. *Clinical Microbiology Reviews*.

[77] Babady N. E., Hall L., Abbenyi A. T., Eisberner J. J., Brown-Elliott B. A., Pratt C. J., McGlasson M. C., Beierle K. D., Wohlfiel S. L., Deml S. M., Wallace R. J., Wengenack N. L. (2010). Evaluation of *Mycobacterium avium* complex clarithromycin susceptibility testing using SLOMYCO sensititre panels and JustOne strips. *Journal of Clinical Microbiology*.

[78] Cook J. L. (2010). Nontuberculous mycobacteria: opportunistic environmental pathogens for predisposed hosts. *British Medical Bulletin*.

[79] Griffith D. E., Aksamit T. R. (2012). Therapy of refractory nontuberculous mycobacterial lung disease. *Current Opinion in Infectious Diseases*.

[80] Jarand J., Levin A., Zhang L., Huitt G., Mitchell J. D., Daley C. L. (2011). Clinical and microbiologic outcomes in patients receiving treatment for *Mycobacterium abscessus* pulmonary disease. *Clinical Infectious Diseases*.

[81] Safdar A. (2012). Aerosolized amikacin in patients with difficult-to-treat pulmonary nontuberculous mycobacteriosis. *European Journal of Clinical Microbiology and Infectious Diseases*.

[82] Döffinger R., Helbert M. R., Barcenas-Morales G., Yang K., Dupuis S., Ceron-Gutierrez L., Espitia-Pinzon C., Barnes N., Bothamley G., Casanova J.-L., Longhurst H. J., Kumararatne D. S. (2004). Autoantibodies to interferon-gamma in a patient with selective susceptibility to mycobacterial infection and organ-specific autoimmunity. *Clinical Infectious Diseases*.

[83] Kampmann B., Hemingway C., Stephens A., Davidson R., Goodsall A., Anderson S., Nicol M., Schölvinck E., Relman D., Waddell S., Langford P., Sheehan B., Semple L., Wilkinson K. A., Wilkinson R. J., Ress S., Hibberd M., Levin M. (2005). Acquired predisposition to mycobacterial disease due to autoantibodies to IFN-*γ*. *The Journal of Clinical Investigation*.

[84] Browne S. K., Zaman R., Sampaio E. P., Jutivorakool K., Rosen L. B., Ding L., Pancholi M. J., Yang L. M., Priel D. L., Uzel G., Freeman A. F., Hayes C. E., Baxter R., Cohen S. H., Holland S. M. (2012). Anti-CD20 (rituximab) therapy for anti-IFN-*γ* autoantibody-associated nontuberculous mycobacterial infection. *Blood*.

[85] Czaja C. A., Merkel P. A., Chan E. D., Lenz L. L., Wolf M. L., Alam R., Frankel S. K., Fischer A., Gogate S., Perez-Velez C. M., Knight V. (2014). Rituximab as successful adjunct treatment in a patient with disseminated nontuberculous mycobacterial infection due to acquired anti-interferon-gamma autoantibody. *Clinical Infectious Diseases*.

[86] Hoefsloot W., van Ingen J., de Lange W. C. M., Dekhuijzen P. N. R., Boeree M. J., van Soolingen D. (2009). Clinical relevance of *Mycobacterium malmoense* isolation in the Netherlands. *European Respiratory Journal*.

[87] Van Ingen J., Totten S. E., Helstrom N. K., Heifets L. B., Boeree M. J., Daley C. L. (2012). *In vitro* synergy between clofazimine and amikacin in treatment of nontuberculous mycobacterial disease. *Antimicrobial Agents and Chemotherapy*.

[88] Wallace R. J., Brown-Elliott B. A., McNulty S., Philley J. V., Killingley J., Wilson R. W., York D. S., Shepherd S., Griffith D. E. (2014). Macrolide/azalide therapy for nodular/bronchiectatic Mycobacterium avium complex lung disease. *Chest*.

[89] Binder A. M., Adjemian J., Olivier K. N., Rebecca Prevots D. (2013). Epidemiology of Nontuberculous Mycobacterial infections and associated chronic Macrolide use among persons with cystic fibrosis. *The American Journal of Respiratory and Critical Care Medicine*.

[90] Wallace R. J., Dukart G., Brown-Elliott B. A., Griffith D. E., Scerpella E. G., Marshall B. (2014). Clinical experience in 52 patients with tigecycline-containing regimens for salvage treatment of Mycobacterium abscessus and Mycobacterium chelonae infections. *Journal of Antimicrobial Chemotherapy*.

[91] Jeon K., Kwon O. J., Nam Y. L., Kim B.-J., Kook Y.-H., Lee S.-H., Young K. P., Chang K. K., Koh W.-J. (2009). Antibiotic treatment of *Mycobacterium abscessus* lung disease: a retrospective analysis of 65 patients. *American Journal of Respiratory and Critical Care Medicine*.

[92] Leber A., Marras T. K. (2011). The cost of medical management of pulmonary nontuberculous mycobacterial disease in Ontario, Canada. *European Respiratory Journal*.

[93] Collier S. A., Stockman L. J., Hicks L. A., Garrison L. E., Zhou F. J., Beach M. J. (2012). Direct healthcare costs of selected diseases primarily or partially transmitted by water. *Epidemiology and Infection*.

